# The Use of Herbal Medicines for the Prevention of Glucocorticoid-Induced Osteoporosis

**DOI:** 10.3389/fendo.2021.744647

**Published:** 2021-11-12

**Authors:** Leiming Zhang, Xiaoli Li, Tianhao Ying, Tian Wang, Fenghua Fu

**Affiliations:** Key Laboratory of Molecular Pharmacology and Drug Evaluation, Ministry of Education, School of Pharmacy, Yantai University, Yantai, China

**Keywords:** glucocorticoid-induced osteoporosis, herb medicine, escin, ginsenoside, glycyrrhizic acid, icariin

## Abstract

Glucocorticoids are drugs that are widely used to suppress inflammation and the activation of the immune system. However, the prolonged use or at high doses of glucocorticoid can result in adverse side effects including osteoporosis, bone loss, and an increased risk of fracture. A number of compounds derived from natural plant sources have been reported to exert anti-inflammatory activity by interacting with the glucocorticoid receptor (GR), likely owing to their chemical similarity to glucocorticoids, or by regulating GR, without a concomitant risk of treatment-related side effects such as osteoporosis. Other herbal compounds can counteract the pathogenic processes underlying glucocorticoid-induced osteoporosis (GIOP) by regulating homeostatic bone metabolic processes. Herein, we systematically searched the PubMed, Embase, and Cochrane library databases to identify articles discussing such compounds published as of May 01, 2021. Compounds reported to exert anti-inflammatory glucocorticoid-like activity without inducing GIOP include escin, ginsenosides, and glycyrrhizic acid, while compounds reported to alleviate GIOP by improving osteoblast function or modulating steroid hormone synthesis include tanshinol and icariin.

## Introduction

Glucocorticoids are drugs that modulate a diverse array of signaling pathways, modifying cognitive signaling, exerting immunosuppressive and anti-inflammatory activity, and preserving normal organ homeostasis and function (1). Since their first clinical deployment in the 1950s, glucocorticoids have been widely adopted and are the most commonly utilized immunosuppressive drug class in the world (2). The prolonged use of glucocorticoids, however, particularly at higher doses, can result in a variety of adverse side effects including arterial hypertension, Cushing’s syndrome, type 2 diabetes mellitus, osteoporosis, and increased susceptibility to infection (3).

Endogenous glucocorticoids regulate key processes including calcium homeostasis in the intestines and kidneys, bone development, and mesenchymal cell differentiation at physiological concentrations. By stimulating mature osteoblasts to increase canonical Wnt protein production, glucocorticoids can promote the activation of β-catenin signaling in mesenchymal progenitor cells such that they differentiate into osteoblasts rather than chondrocytes or adipocytes, thus favoring osteogenesis. In osteoblasts, Wnt signaling also leads to the expression of osteoprotegerin (OPG), which suppresses osteoclastogenesis to maintain bone homeostasis (4). At very high doses, however, glucocorticoids can negatively impact bone integrity through a range of mechanisms, with GIOP having first been described in individuals with Cushing’s disease expressing excess endogenous glucocorticoid levels (5).

Owing to their potent anti-inflammatory and immunomodulatory activity, glucocorticoids are widely used in clinical contexts. However, their prolonged use can lead to adverse outcomes including glucocorticoid-induced osteoporosis (GIOP), which is the most common secondary cause of osteoporosis and an important iatrogenic risk to patients in many contexts (6). Such osteoporosis has been reported in patients with chronic inflammatory diseases including inflammatory bowel disease, chronic obstructive pulmonary disease, and systemic lupus erythematosus (SLE) (7). Most SLE patients undergo chronic glucocorticoid treatment, and one Dutch study with a 6-year follow-up period detected a dose-dependent association between the use of glucocorticoids and lumbar spine bone loss (8). Similarly, a cohort study of individuals between the ages of 18 and 64 undergoing glucocorticoid treatment for a range of disorders found that higher doses, longer treatment durations, and continuous use were associated with the highest fracture risk (9). Sustained treatment with prednisone (10 mg/d) for over 90 days was associated with 7- and 17-fold increases in the risk of hip and vertebral fractures (9).

**Figure 1:**
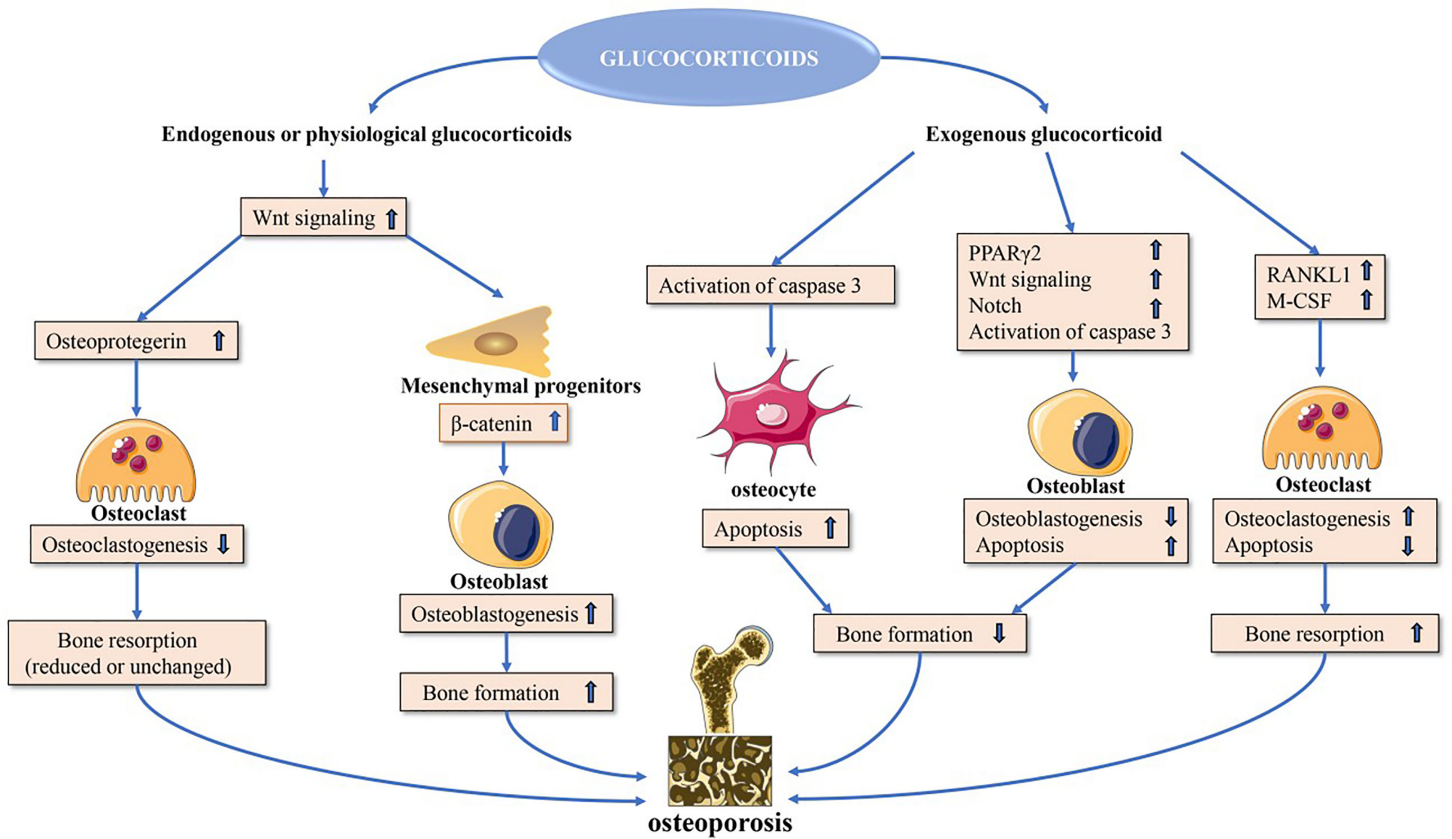
Direct glucocorticoid effects on bone. Endogenous or physiological glucocorticoids stimulate mature osteoblasts to produce canonical Wnt proteins, which activate the β-catenin signaling cascade in mesenchymal progenitor cells and promote them to differentiate towards osteoblasts. These actions favor bone formation. Additionally, Wnt signaling in osteoblasts and osteocytes promotes osteoprotegerin expression, which in turn inhibits osteoclast formation resulting in decreased or unchanged bone resorption. Exogenous glucocorticoids negatively affect osteoblast and osteocyte function. In osteoblasts and osteocytes, increased PPARγ2 and Notch target gene expression and decreased wnt signaling contribute to decreased osteoblastogenesis, and activation of caspase 3 results in increased osteoblast and osteocyte apoptosis. Glucocorticoids induce upregulation of expression of RANKL and M-CSF, which leads to increased osteoclastogenesis and osteoclast lifespan. PPARγ2, peroxisome proliferator-activated receptor-γ2; RANKL, receptor activator of nuclear factor-κB ligand; M-CSF, macrophage colony-stimulating fact.

Glucocorticoids can modulate bone biology *via* a number of different mechanisms (**Figure 1**), suppressing osteogenesis and promoting the apoptotic death of osteoblasts and osteocytes (10). Additionally, these drugs can increase the number of osteoclasts and enhance their function, resulting in an overall increase in osteoclast lifespan (11).

Osteoblast signaling pathways that can be directly impacted by glucocorticoid exposure include the peroxisome proliferator-activated receptor γ2 (PPARγ2) (12), CCAAT/enhancer-binding protein-α (C/EBPα) (13), adipocyte protein 2 (aP2) (14), and canonical WNT signaling pathways (15). Glucocorticoids promote PPARγ2, C/EBPα, and aP2 upregulation, leading precursor cells to preferentially differentiate into adipocytes instead of osteoblasts, thereby leading to a decrease in overall osteoblast numbers (12–15). Glucocorticoids also increase the expression of inhibitory molecules including sclerostin in the WNT-β-catenin signaling pathway while simultaneously inhibiting the expression of WNT16 in a dose- and time-dependent fashion, further contributing to reduced osteoblastogenesis and bone loss (16, 17).

The receptor activator of nuclear factor-κB ligand (RANKL)-osteoprotegerin (OPG) pathway is also amenable to modulation by glucocorticoids, which increase RANKL production and suppress OPG *mRNA* expression (18–20). Glucocorticoids can also enhance Notch signaling in osteoblasts and osteocytes, leading to increased Notch target gene expression including hairy and enhancer of split (Hes) and Hes-related with YRPW motif (Hey), which are repressive transcription factors that have the potential to mediate the impairment of osteoblast functionality and consequent reductions in osteogenesis (21, 22).

Glucocorticoid-induced apoptosis is linked to the enhanced activity of effector proteins including caspase 3, 7, and 8 downstream of the pro-apoptotic Bim and Fas/FasL death receptor pathways (10). Glucocorticoids can also stabilize GSK-3β activity to induce osteoblast apoptosis.

Glucocorticoids can impact osteoblasts to increase the RANKL : OPG ratio, thereby promoting osteoclast differentiation and maturation such that the overall rate of bone resorption increases. This effect can be further exacerbated by the ability of glucocorticoid treatment to induce the production of macrophage colony stimulating factor (M-CSF), which is released from osteoblasts and enhances the differentiation and activity of osteoclasts (23). The long-term impact of glucocorticoids on osteoclast function, however, is less certain, with multiple reports indicating that these compounds can interfere with the osteoclast cytoskeleton such that the activity of these cells may be increasingly impaired even as their longevity increases (24–26).

## The Impact of Herbal Medicines on Glucocorticoid- Induced Osteoporosis

Many studies have shown that herbal medicines can significantly increase bone density and improve clinical findings in GIOP patients, thus serving as novel tools for the treatment and/or prevention of this debilitating glucocorticoid-related complication (27–29).

### Herbal Medicines Exert Glucocorticoid-Like Anti-Inflammatory Activity Without Inducing GIOP

A range of herbal compounds have been suggested to mediate anti-inflammatory activity by signaling through the glucocorticoid receptor, likely owing to their structural similarity to glucocorticoids. Notably, these compounds seem to be able to mediate these effects without a significant risk of negative glucocorticoid-related side effects such as GIOP.

#### Escin

Escin is a natural mixture of triterpene saponins extracted from the seeds of Aesculus chinensis Bge. or Aesculus wilsonii Rehd. Escin has been reported to exhibit pharmacological effects similar to those associated with glucocorticoid administration. For example, oral escin (5 and 10 mg/kg, p.o.) intake has been found to suppress carrageenan-induced paw edema and to inhibit prostaglandin E2 (PGE2) production (30). Notably, when compared with glucocorticoids, escin (2 mg/kg, i.v.) has been shown not to induce thymic or splenic immune cell apoptosis in mice, nor does it promote the enhanced secretion of endogenous corticosterone (31). Zhang et al. found that the sustained administration of escin (0.45 and 0.9 mg/kg for a period of 10 days, i.v.) in the context of post-surgical bone fracture healing has no adverse impact on wound or bone healing processes (32). There is also evidence that glucocorticoids and escin (2 mg/kg, i.v.) exhibit synergistic anti-inflammatory activity when administered *in vitro* and *in vivo* at low doses, suggesting at least partial overlap in the pharmacological pathways impacted by these compounds (33). Combination glucocorticoid and escin(5 and 10 mg/kg for a period of 16 days, i.g.) treatment can significantly decrease synovial inflammatory infiltration, synovial hyperplasia, and bone erosion in a rat model of adjuvant-induced arthritis (AIA) rats while reversing some of the adverse effects of glucocorticoid treatment alone such as reductions in boy weight and increases in the spleen index relative to untreated rats (34). Administering escin (10 mg/kg for a period of 14 days, p.o.) together with a low dose of dexamethasone (Dex) has been shown to markedly suppress paw swelling, joint pathology, arthritic index scores, and immune organ pathology in an animal model, all while reducing the necessary Dex dose and thus decreasing the rate of adverse effects associated with Dex administration (35). The anti-edema and anti-inflammatory properties of escin may be attributable to its ability to bind to the glucocorticoid receptor (GR), consistent with glucocorticoid-like activity (36). Escin (1.8 and 3.6 mg/kg, i.v.) may additionally augment the antioxidant capacity of tissue in the context of lipopolysaccharide (LPS)-induced acute lung injury (ALI) and endotoxin-induced liver injury by suppressing the production of inflammatory compounds including NO, TNF-α, and IL-1β while simultaneously promoting GR upregulation in the liver and lungs (37, 38).

#### Ginsenosides

Ginsenosides are the primary active ingredients isolated from ginseng, and they have been reported to exhibit anti-inflammatory activity *in vitro* and *in vivo* owing to their structural similarity to steroid hormones. Compound K is a ginsenoside that has, *in vitro*, been shown to suppress TNF-α-induced fibroblast-like synoviocyte (FLS) migration, proliferation, and secretion, consistent with joint-protective activity (80 mg/kg for a period of 14 days, i.g.) (39). In a rat model of myocardial infarction (MI), ginsenoside Rg3 (30 mg/kg for a period of 7 days, i.g.) reduces inflammation *via* the inhibition of the NF-κB pathway (40). Combining the ginsenosides Rh1 (20 mg/kg, i.p.) and Rg2 (20 mg/kg, i.p.) can suppress LPS-induced tissue damage and inflammation by interfering with the ability of LPS to bind to and trigger the activation of TLR4 (41). Ginsenoside Rb1 (10 and 20 mg/kg, i.p.) markedly alleviates LPS- or cantharidin-induced acute kidney injury, LPS-induced septicemia, and dimethyl benzene-induced ear edema in mice (42). Ginsenoside treatment is also not associated with any significant adverse reactions. In mice overexpressing TNF-α, ginsenoside Rg1 (20 mg/kg, i.g.) can prevent bone erosion, inhibit synovial inflammation, and reduce serum levels of both IL-6 and TNF-α, and treatment for 12 weeks with ginsenoside Rg1 was not associated with any liver or kidney damage (43). Ginsenoside Rd (10 mg/kg, i.p.) can suppress ischemia-induced microglial activation and inhibit proinflammatory cytokine production while inducing fewer severe side effects as compared to glucocorticoids (44). Importantly, these ginsenosides can also work in synergy with glucocorticoids to inhibit inflammation. For example, combining corticosterone with low concentrations of Rg1 can suppress the LPS-induced production of NO and TNF-α by RAW264.7 macrophages while simultaneously promoting GR upregulation (45). Ginsenosides can also shape cellular responses in a GR-mediated manner, as in the case of Rg1 (12.5 mg/kg, i.p.), which suppresses LPS-induced NF-κB nuclear translocation and inflammatory cytokine production in a GR-dependent fashion. Notably, Rg1 (20 mg/kg for a period of 21 days, i.g.) has no adverse impact on murine osteoblast differentiation or proliferation (46). In a murine collagen-induced arthritis (CIA) model system, the ginsenoside Rh1 (10 mg/kg for a period of 10 days, i.p.) was also able to augment the anti-inflammatory activity of Dex by enhancing GR expression and binding without inducing hyperglycemia (47). Ginsenoside CK (112 mg/kg for a period of 24 days, i.g.) can also activate GR to suppress β-arrestin2 expression, thereby inhibiting inflammation (48).

#### Glycyrrhizic Acid and Glycyrrhetinic Acid

Glycyrrhizic acid (also known as glycyrrhizin) is the primary glycoside derivative of licorice, and it has been ascribed a range of anti-inflammatory activities. By suppressing signaling through the Smad3 and MAPK pathways, for example, glycyrrhizin (30 and 100 mg/kg for a period of 28 days, i.g.) has been shown to reduce the severity of bleomycin-induced inflammation and pulmonary fibrosis in mice (49). Glycyrrhizin (10 mg/kg for once every day in the first 3 weeks following by given once every 3 days until the twelfth week, intra-articular knee injection) treatment can also alleviate inflammation and the degeneration of cartilage tissue in a rat model of osteoarthritis *via* regulating the TLR4/NF-κB and HMGB1 pathways (50). *In vivo*, glycyrrhizic acid undergoes hydrolysis to yield glycyrrhetinic acid, which is structurally similar to steroid hormones such that it is able to exert a range of biological effects including glucocorticoid-like anti-inflammatory activity through interactions with steroid hormone receptors and metabolic enzymes. For example, in a murine ALI model system, glycyrrhetinic acid (10, 20 and 40 mg/kg for a period of 7 days, i.g.) was able to reduce injury severity by suppressing NLRP3 inflammasome activation through the ROS-PI3K/AKT pathway (51). Glycyrrhetinic acid (40 μM) may also be hepatoprotective in the context of chronic liver inflammation, functioning by suppressing the phosphorylation of IκBα phosphorylation and the nuclear translocation of p65 so as to reduce iNOS expression, thus alleviating inflammation (52). Glycyrrhetinic acid and glycyrrhizic acid can interact with GR as ligands, modulating glucocorticoid resistance can also prevent inflammation by disrupting the GR-HSP90 (53, 54). As a relatively weak glucocorticoid-like drug, glycyrrhizic acid can enhance the effects of glucocorticoids while antagonizing the adverse effects associated with high-dose glucocorticoid treatment. Licorice (75 mg/kg for a period of 5 days) can also suppress 11 beta-hydroxysteroid dehydrogenase mRNA expression while potentiating glucocorticoid activity (55). Therefore, glycyrrhizic acid and glycyrrhetinic acid seem to be able to exert glucocorticoid-like anti-inflammatory activity without a significant risk of negative glucocorticoid-related side effects such as GIOP.

### Herbal Medicines Capable of Inhibiting or Treating GIOP

#### Icariin

Icariin is the main active ingredient of epimedium, which is a natural compound that has been increasingly studied in the context of osteoporosis treatment and prevention, as it has been shown to simultaneously suppress bone resorption and expedite bone formation (56). Icariin (125 mg/kg for a period of 14 days, i.g.) can promote primary osteoblast maturation and associated bone remodeling, inducing osteoblast mineralization and the expression of key markers of terminal differentiation such as alkaline phosphatase (ALP) and type I collagen (57–60). Icariin(0.1 μM) also exhibits robust anti-apoptotic activity, promoting BMSC proliferation and osteogenic differentiation *via* Wnt/β-catenin pathway activation (61).

With respect to the symptoms of GIOP, icariin (5 μM for a period of 48 h) can enhance trabecular bone density in the context of glucocorticoid exposure, promoting osteogenic differentiation *via* the suppression of Notch signaling (62). Through the enhancement of autophagic activity, icariin (50 mg/kg for a period of 30 days, i.p.) can reduce OVX-induced bone loss in animal model systems (63), in addition to disrupting the Dex-induced apoptotic death of osteocytes (58). Icariin can also activate the ERK and ER pathways to control bone homeostasis, promoting OPG expression and Wnt pathway activation. Inhibiting osteoclastogenesis is at least partially responsible for the anti-osteoporotic activity of icariin and compounds derived therefrom. The levels of the osteoclast differentiation marker tartrate-resistant acid phosphatase (TRAP) are reduced in a dose-dependent manner when osteoclast precursor cells are treated with icariin (10 nM, every 3 days) (64). Icariin (10 μM) is also able to directly suppress RANKL-induced hemopoietic cell differentiation into osteoclasts (65). In addition to regulating osteoclastogenesis, icariin (50 and 100 μM) can arrest cell cycle progression in osteoclast precursors, thereby inducing their apoptotic death (66). It can further reverse deleterious Dex-induced trabecular phenotypes while stimulating bone remodeling, increasing bone calcium, OCN, and FGF-23 levels while reducing the levels of bone resorption markers including CTX and TRAP-5b. Indeed, in GIOP model mice, icariin (100 mg/kg for a period of 6 or 12 weeks, p.o.) treatment has been shown to protect against bone degeneration, hypercalciuria, and hypocalcemia (67). As such, icariin may be a valuable tool for use in the induction of bone regeneration owing to its potent osteogenic bioactivity.

Many clinical studies have shown that Chinese medicine containing epimedium has achieved good clinical effects in the treatment of GIOP patients. Hugu Capsules (comprised of epimedium, polygonum multiflorum, rehmannia glutinosa and other traditional chinese medicines) can significantly increase the bone mass of 51 patients with GIOP, improve bone turnover, and relieve pain (68). Through observation of 50 GIOP patients, Wu et al. found that taking Xianling Gubao capsule while using glucocorticoids treatment can increase the BMD of the patient’s lumbar spine and proximal femur, thereby reducing the incidence of osteoporotic fractures and having fewer adverse reactions (69). Through clinical observation of 66 patients with GIOP, Shi et al. found that Bugu Capsules (including epimedium) can significantly reduce the impact of OP caused by glucocorticoids, reduce blood calcium, parathyroid hormone levels, and increase bone density (70).

#### Tanshinones

Tanshinone IIA, extracted from Salvia miltiorrhiza Bunge, is a perennial herbal plant widely used as a folk remedy in Asian countries. Several studies have proved that Tanshinone IIA possesses many biological activities, such as anti-inflammatory, free-radical scavenging abilities, antioxidant properties, liver protection, and anti-cancer properties. Tanshinones are compounds that can also simultaneously inhibit osteoclastogenesis and bone resorption while promoting more robust bone formation with concomitant osteoblastogenesis. These tanshinones (2, 5 μg/ml) suppress osteoclast development through the disruption of RANKL-mediated NF-κB, MAPK, Akt, and M-CSF/c-Src signaling pathway activation (71, 72). Tanshinone IIA, for example, can inhibit osteoclastogenesis through the inhibition of RANKL-induced c-Fos and NFAT c1 (71), with Tanshinone IIA (20 μg/mL for a period of 30 min) pretreatment reportedly reducing the fusion, actin ring formation, and resorptive activity of osteoclasts in a co-culture system containing M-CSF and RANKL-treated calvarial osteoblasts and BMCs (73). Mechanistically, Tanshinone IIA (10 μg/mL) can function as a selective COX-2 inhibitor to suppress PGE2 and to thereby modulate OPG and RANKL expression (74), all of which are related to osteoclast function. Tanshinones (1 μM for a period of 24 h) can also disrupt the apoptotic death of osteoblasts and consequent osteoporosis observed upon glucocorticoid treatment by inactivating Nox4 (75). In osteoporosis model mice, Tanshinone IIA (10 mg/kg for a period of 6 weeks, p.o.) was able to decrease the incidence of fractures and severe osteopenia while augmenting bone strength, mineral levels, and collagen in the bone matrix (76). Tanshinone (10 mg/kg for a period of 21 days, i.v.) was also able to upregulate phosphoglycerate dehydrogenase and to thereby suppress OVX-induced osteoporosis and BMSC senescence (77). Tanshinone can alleviate the adverse effects of Dex treatment and consequence cellular injuries such as caspase-9-dependent apoptosis, increased cytosolic cytochrome c and Nox levels, and increased ROS generation (75). Current preclinical evidence suggests that these Tanshinones preserve skeletal integrity primarily by suppressing bone resorption and osteoclast formation, underscoring their potential value for the treatment of GIOP.

Many other herbal medicines have also been found to reduce GIOP incidence or severity through a range of mechanisms. For example, celastrol can suppress GIOP incidence in rats by modulating the Wnt and PI3K/AKT signaling pathways (78), while KRG can induce the apoptotic death of osteoblasts, highlighting its potential therapeutic utility as a tool to delay the onset of osteoporosis (79). Osthole has been shown to prevent Dex-induced osteoporosis in female rats, potentially by normalizing hormone and cytokine homeostasis through increases in TGF-β1 production (80) (**Table 1**).

**Table 1:** Herbal medicines capable of inhibiting or treating GIOP.

Origin	Main components	*In vitro*		*In vivo*		Mechanism of bone protection
		Cells	Dosage	Animal	Dosage and administration route	
*Celastrus genus of the Celastraceae family*	Celastrol (78)	–	–	Male C57BL/6J mice	1 mg/kg, per day for 12 weeks, i.m.	Activating Wnt signaling pathway
*Daphne odora* var. *marginatai*	Daphnetin (81)	MC3T3-E1 cells	20 μM for 48 h	Male SD rats	i.m., i.p.	
*Herba Cistanches*	Echinacoside (82)	MC3T3-E1 cells	10 mg/l for 48h	–	–	Induction of osteoblast apoptosis
*Ginkgo Biloba*	Ginkgo biloba extract (83)	–	–	Female Wistar rats	28, 56 mg/kg, per day for 20 days or 30 days, i.g.	
Red Ginseng	Red Ginseng (79)	MC3T3-E1 cells	250, 500, 1000 μg/mL for 48h	–	–	
*Cnidium monnieri (L.) Cusson*	Osthole (80)	–	–	Female SD rats	10, 20 mg/kg, per day for 8 weeks, i.m.	Regulating TGF-β/Smad signaling
*Rhizoma gastrodiae*	Gastrodin (84)	MC3T3-E1 cells	1, 5 μM for 48 h	Female SD rats	1, 5 mg/kg, per day for 60 days, i.g.	Upregulating expression of BMP
*Myrica rubra Sieb. et Zucc.*	Myricetin (85)	MC3T3-E1 cells	20 μM	Male SD rats	2.5 mg/kg,once every other day for a period of 5 weeks, i.p.	
*Curcuma longa*	Curcumin (86)	–	–	Male C57BL/6J mice	200 mg/kg per day for 12 weeks, i.g.	Inhibiting the activity of RANKL/RANK signaling
Chansu	Gamabufotalin (87)	BMMs	100, 150 nM for 3-5 days	–	–	
*Piper sarmentosum Roxb.*	Piper sarmentosum (88)	–	–	Male SD rats	125 mg/kg	Inhibiting the activity of 11β-HSD1
*Achyranthes bidentata Bl.*	β-ecdysone (89)	BMSC	10^-7^ M for 8 h	Male Swiss-Webster mice	0.5 mg/kg	Inhibiting the autophagy produced by osteoclasts
*Pueraria Lobata*	Total Flavones of Pueraria Lobata (90)	–	–	Female SD rats	100, 200 mg/kg, per day for 12 weeks, i.g.	Promoting bone matrix formation
*Pueraria pseudo-hirsuta TANG et WANG*	Chilk extracts (91)	–	–	Female wistar rats	200mg/kg, per day for 6 months, i.g	Decreasing sex hormone levels
*Lycium chinense Miller*	Lycium barbarum polysaccharide (92)	–	–	Wistar rats	2.6 g/kg, per day for 12 weeks, i.g.	Regulating calcium and phosphorus metabolism
*Salvia miltiorrhiza Bunge*	Salvianolic acid B (93)	–	–	Male SD rats	40, 80 mg/kg, per day for 12 weeks, p.o.	Regulating lipid metabolism balance

## Conclusion

Much like other hormone molecules, glucocorticoids can exert a range of effects on tissues and organs when employed at physiological and pharmacological doses. While awareness of osteoporosis and other risks associated with prolonged or high doses glucocorticoid use is growing, GIOP remains underdiagnosed and inadequately treated. Herbal medicines characterized to date have been shown to treat GIOP through two primary mechanisms, with some exerting glucocorticoid-like activity without a risk of adverse reactions, and the others treating GIOP through mechanisms including the regulation of Wnt signaling pathway, the induction of osteoblast apoptosis, and the inhibition of RANKL/RANK signaling.

However, Further clinical studies of these herbal medicines are needed to demonstrate prevention properties in GIOP patients. For example, sodium aescinate has been widely used in clinic to treat traumatic and inflammatory edema, etc. A randomized, parallel, controlled clinical trial can be conducted to evaluate the anti-inflammatory efficacy combined with glucocorticoids, as well as the side effects, GIOP.

## Author Contributions

LZ and FF conceived the idea. XL and TY drafted the manuscript. LZ, TW, and FF supervised the process and contributed to editing. All authors contributed to the article and approved the submitted version.

## Funding

This work was supported by the National Science Foundation of China (No. 81973547).

## Conflict of Interest

The authors declare that the research was conducted in the absence of any commercial or financial relationships that could be construed as a potential conflict of interest.

## Publisher’s Note

All claims expressed in this article are solely those of the authors and do not necessarily represent those of their affiliated organizations, or those of the publisher, the editors and the reviewers. Any product that may be evaluated in this article, or claim that may be made by its manufacturer, is not guaranteed or endorsed by the publisher.
