# Supplementary material for: The Use of Herbal Medicines for the Prevention of Glucocorticoid-Induced Osteoporosis
Source: Front Endocrinol (Lausanne). 2021 Nov 12;12:744647. doi: 10.3389/fendo.2021.744647 (PMC8633877; doi:10.3389/fendo.2021.744647)
Supplement: Supplementary file 1 [file Table_1.docx]

Supplement table

| **Origin** | **Main components** | **Mechanism of anti-inflammatory** | **Duration** | **Glucocorticoid-like side effect** |
| --- | --- | --- | --- | --- |
| *Aesculus wilsonii Rehd.* | Escin | 1. Activating GR  2. Down-regulating levels of inflammation mediators (TNF-α, IL-1β and NO) and 11β-HSD2 expression in liver, up-regulated GR expression | 24 h | NO |
| *Panax ginseng C. A. Meyer* | Ginsenoside CK | Activating GR to suppress β-arrestin2 expression | - | NO |
|  | Ginsenoside Rg3 | Reducing inflammation via the inhibition of the NF-κB pathway |  |  |
|  | Ginsenoside Rh1 | Interfering with the ability of LPS to bind to and trigger the activation of TLR4 |  |  |
|  | Ginsenoside Rg2 |  |  |  |
|  | Ginsenoside Rb1 | Modulating toll-like receptor 4 dimerization and NF-kB/MAPKs signaling pathways |  |  |
|  | Ginsenoside Rg1 | Regulating the GR-dependent BMP-2/Smad pathway |  |  |
|  | Ginsenoside Rd | Inhibiting proinflammatory cytokine production |  |  |
| *Glycyrrhiza uralensis Fisch.* | Glycyrrhizic acid | 1.Suppressing signaling through the Smad3 and MAPK pathways  2.Regulating the TLR4/NF-κB and HMGB1 pathways | - | NO |
|  | Glycyrrhetinic acid | 1.Suppressing NLRP3 inflammasome activation through the ROS-PI3K/AKT pathway  2.Suppressing the phosphorylation of IκBα phosphorylation and the nuclear translocation of p65 so as to reduce iNOS expression |  |  |
